# Tissue Distribution of Berberine and Its Metabolites after Oral Administration in Rats

**DOI:** 10.1371/journal.pone.0077969

**Published:** 2013-10-31

**Authors:** Xiang-Shan Tan, Jing-Yi Ma, Ru Feng, Chao Ma, Wen-Jing Chen, Yu-Peng Sun, Jie Fu, Min Huang, Chi-Yu He, Jia-Wen Shou, Wen-Yi He, Yan Wang, Jian-Dong Jiang

**Affiliations:** State Key Laboratory of Bioactive Substance and Function of Natural Medicines, Institute of Materia Medica, Chinese Academy of Medical Sciences and Peking Union Medical College, Beijing, China; Biological Research Centre of the Hungarian Academy of Sciences, Hungary

## Abstract

Berberine (BBR) has been confirmed to have multiple bioactivities in clinic, such as cholesterol-lowering, anti-diabetes, cardiovascular protection and anti- inflammation. However, BBR’s plasma level is very low; it cannot explain its pharmacological effects in patients. We consider that the *in vivo* distribution of BBR as well as of its bioactive metabolites might provide part of the explanation for this question. In this study, liquid chromatography coupled to ion trap time-of-flight mass spectrometry (LC/MS^n^-IT-TOF) as well as liquid chromatography that coupled with tandem mass spectrometry (LC-MS/MS) was used for the study of tissue distribution and pharmacokinetics of BBR in rats after oral administration (200 mg/kg). The results indicated that BBR was quickly distributed in the liver, kidneys, muscle, lungs, brain, heart, pancreas and fat in a descending order of its amount. The pharmacokinetic profile indicated that BBR’s level in most of studied tissues was higher (or much higher) than that in plasma 4 h after administration. BBR remained relatively stable in the tissues like liver, heart, brain, muscle, pancreas etc. Organ distribution of BBR’s metabolites was also investigated paralleled with that of BBR. Thalifendine (M1), berberrubine (M2) and jatrorrhizine (M4), which the metabolites with moderate bioactivity, were easily detected in organs like the liver and kidney. For instance, M1, M2 and M4 were the major metabolites in the liver, among which the percentage of M2 was up to 65.1%; the level of AUC _(0-t)_ (area under the concentration-time curve) for BBR or the metabolites in the liver was 10-fold or 30-fold higher than that in plasma, respectively. In summary, the organ concentration of BBR (as well as its bioactive metabolites) was higher than its concentration in the blood after oral administration. It might explain BBR’s pharmacological effects on human diseases in clinic.

## Introduction

**Figure 1:**
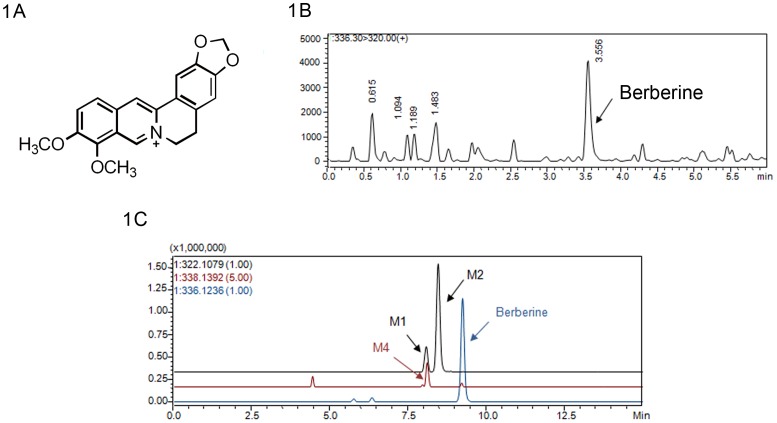
BBR mass spectrographic behavior and its metabolism in tissues. 1A Structure of BBR 1B Mass spectrographic behavior of BBR 1C Representative EIC chromatogram of BBR and its metabolites in liver (8 h) in rat.

Berberine (BBR, structure was shown in Fig.1A), an isoquinoline alkaloid, is an important bioactive component isolated from the rhizome of *Coptis chinensis* (“Huang-Lian” in Chinese) of Ranunculaceae family. BBR has been used as antimicrobial for a long history in China. Besides its anti-microbial effect [1]–[4], BBR has been used in clinic for hyperlipidemia [5], diabetes [6], neuroprotective [7] and cardiovascular diseases [8], suggesting its potential in future. BBR showed anti-hyperlipidemia effect of lowering total cholesterol (TC), triglyceride (TG), low-density-lipoprotein cholesterol (LDL-c) levels in patients [5]. The cholesterol-lowering mechanism of BBR was different from that of statins. Statins up-regulate the low density lipoprotein receptor (LDLR) via inhibiting cholesterol synthesis, while BBR elevates the LDLR expression by stabilizing LDLR message ribonucleic acid (mRNA). Although BBR itself is the most active form, its metabolites remain active with 30–70% activity [9]. For instance, for LDLR up-regulation and adenosine 5′-monophosphate-activated protein kinase (AMPK) activation, berberrubine and thalifendine are the two active metabolites, but with reduced potency [9].

Besides hyperlipidemia, BBR has also been reported to be effective in anti-diabetes. This was firstly discovered by Chen QM et.al in 1986 [10]. It has been confirmed that the mechanisms of anti-diabetes involve in activation of AMPK [11] and improvement of insulin sensitivity [12]. In addition, it is reported that BBR improves fasting blood glucose (FBG) by direct inhibition of gluconeogenesis in liver [13]. In the clinical study, BBR significantly lowered FBG, hemoglobin A_1C_, triglyceride, and insulin levels in patients with type 2 diabetes mellitus (T2DM) [14]. Further more, BBR also could exert renoprotective effects on diabetic nephropathy in mice [15].

Being able to permeate the central nervous system (CNS) is the prerequisite of neuroprotective agents. Due to its character of entering CNS [16], BBR has shown its potential in the treatment of nervous system diseases, such as neurodegenerative disease and neurotrosis, Alzheimer’s disease (AD) [17], toxicity-induced neuronal injure [3], neuro-inflammation [4], cerebral hypoxia and ischemic brain injury [18]. Moreover, researches on BBR have shown its effects on cardiovascular diseases by reducing cardiotoxicity, improving cardiac dysfunction, arrhythmia and atherosclerosis. [18]–[20].

In contrast to its significant pharmacological effects in clinic, the plasma level of BBR is very low [21]. Absolute bioavailability of BBR was reported to be less than 1% [22]–[24] based on *C*_max_ calculation in blood [25]. Efforts have been carried out to explain the discrepancy. In reviewing our BBR study records and related literature from other groups, we consider that it is necessary to analyze the distribution of BBR as well as its bioactive metabolites in main organs or tissues, and compare that with BBR’s level in blood, after oral administration.

For the technique part, there have been some detection methods used for BBR, such as high performance liquid chromatography-ultraviolet detector (HPLC-UV) [26], high performance liquid chromatography-fluorescence detection (HPLC-FD) [27], field desorption mass spectrometry (FDMS) [28], gas chromatography chemical ionization mass-spectrometry (GC-CI-MS) [29] and high performance liquid chromatography–mass spectrometry (HPLC-MS) [9], [22], [30], but these methods were not easy to perform for both quantitative and qualitative analysis of BBR and its trace metabolites in biological samples. In recent years, LC/MS^n^-IT-TOF has become a powerful tool for qualitative analysis of complex constituents in natural products and metabolites based on abundant information of the fragmentation in multiple-stage mass spectrum and accurate molecular weight [31]–[34]. In this study, both LC/MS^n^-IT-TOF and tandem mass spectrometry (LC-MS/MS) were firstly used to investigate the tissue distribution and pharmacokinetics of BBR in rats and simultaneously achieve a full structure confirmation of BBR and metabolites from IT-TOF and accurately quantitative analysis in mode of multiple reactions monitoring (MRM) by LC-MS/MS. The goal was to illustrate a tissue distribution profile of BBR and its metabolites for oral administration of BBR.

## Materials and Methods

### Chemicals and Reagents

BBR and palmatine were purchased from J&K Scientific Ltd (Beijing, China) and the National Institute for the Control of Pharmaceutical and Biological Products (Beijing, China), respectively. Berberrubine, thalifendine, demethylenberberine and jatrorrhizine were supplied from Chengdu Must Bio-technology Co., Ltd (Chengdu, China). The purity of all standards above was more than 98%. High–performance liquid chromatography-grade acetonitrile was obtained from J&K Scientific Ltd. Deionized distilled water was Wa ha ha purified water (Hangzhou Wa ha ha Group Co., Ltd, China). The other chemical reagents were of the highest grades available from Sinopharm Chemical Reagent Co., Ltd (Beijing, China).

### Animals

Sprague-Dawley rats (male, 180–220 g, 6–7 weeks) for the following pharmacokinetic study of plasma (section 7 Preparation of tissue samples) and Preparations of tissue samples (section 8 Identification of BBR metabolites in tissues) were obtained from the Department of Laboratory Animal Science, Chinese Academy Medical Sciences (Beijing, China), and housed with free access to food and water. The animals were maintained on a 12-h light/dark cycle (light on from 8∶00 AM to 8∶00 PM) at ambient temperature (22–24°C) with 45% relative humidity. Rats were fasted for 12 h before all experimental studies. Research was conducted in accordance with all institutional guidelines and ethics and approved by the Laboratories Institutional Animal Care and Use Committee of the Chinese Academy of Medical Sciences and Peking Union Medical College.

Six male SD rats were oral administrated with BBR (200 mg/kg), which was dissolved by saline into liquid suspension, with concentration of 40 mg/ml. Blood samples (0.5 ml) were obtained from posterior orbital venous plexus to a heparinized tube at 0, 0.25, 0.5, 1, 1.5, 2, 3, 4, 6, 8, 12, 24 h, respectively, and then centrifuged at 5000 r/min for 5 min. The plasma was collected and stored in −20°C.

Sixty-six male SD rats (n = 6 for each time point), after oral administration of BBR (200 mg/kg), were sacrificed by cervical dislocation for the collection of liver, kidneys, muscle, lungs, brain, heart, pancreas and fat at 0.25, 0.5, 1, 2, 4, 6, 8, 12, 24 and 48 h, respectively. All tissue samples were stored in −20°C.

### Instruments

Chemical Structure confirmation of BBR and its metabolites was performed by a Shimadzu high-performance liquid chromatography coupled to ion trap time-of-flight mass spectrometry (LC/MS^n^-IT-TOF, Shimadzu Cooperation, Japan) with an Alltima C_18_ column (150 mm×4.6 mm, 5 µm *i.d*., Alltech Cooperation, USA). Samples were eluted through the column with a gradient of water-formic acid (100∶0.5, v/v) and acetonitrile (0 min, 85∶15; 12 min, 65∶35; 15 min, 85∶15) at a flow rate of 0.8 ml/min at 40°C.

A Shimadzu triple quadrupole mass spectrometry (LC-MS/MS 8040, Shimadzu Cooperation, Japan) was used for separation and determination of biological samples for pharmacokinetics and tissue distribution. LC separation was achieved using a Shim-pack XR-ODS II column (75 mm×3 mm, 2.3 µm *i.d.*, Shimadzu Cooperation, Japan) maintained at 40°C. The mobile phase consisted of the same solvents as the one above with a linear gradient elution (0 min, 85∶15; 5 min, 20∶80; 6.0 min, 20∶80; 6.01 min, 85∶15; 9 min, 85∶15) with a flow rate of 0.5 ml/min during the whole gradient cycle. An injection time was within 9 min. Shimadzu LCMS solutions (Version 5.50.316 and 3.60.361) were used for data acquisition and processing.

### Preparation of Plasma Sample

Plasma (100 µl) was extracted with 1.25 ml ethyl ether after addition of 10 µl internal standard (250 ng/ml of palmatine) and 50 µl 0.5 M sodium hydroxide solution. Following centrifugation and separation, the organic phase (1 ml) was evaporated to dryness in a 40°C water bath by sample concentrator (Nitrogen blowing instrument). The residue was reconstituted with 100 µl mobile phase (a mixture of acetonitrile and 0.5% formic acid with ratio of 1∶4). An aliquot of 10 µl was injected into the LC-MS/MS 8040 system.

### Methodology Validation

Stock solution of BBR was prepared by dissolving 5.0 mg in 50 ml methanol producing a concentration of 100 µg/ml and stored at 4°C. Working solutions were prepared at a series of concentration of 0.5, 1, 5, 10, 50, 100 and 500 ng/ml by diluting the stock solution with methanol. A solution containing internal standard (250 ng/ml, palmatine) was also prepared in methanol. The method validation assays were carried out according to the currently accepted Chinese State Food and Drug Administration (SFDA) bioanalytical method validation guidance. Selectivity, linearity, precision, accuracy, extraction recovery, matrix effect and stability were assayed respectively.

### Pharmacokinetic Study of Plasma in Rat

100 µl of each plasma sample, obtained as section 2 (Animals), was added with 10 µl internal standard and 50 µl 0.5 M sodium hydroxide solution. Following centrifugation and separation were performed as section 4 (Preparation of plasma sample). The residue was reconstituted with 100 µl mobile phase (a mixture of acetonitrile and 0.5% formic acid with ratio of 1∶4). An aliquot of 10 µl was injected into the LC-MS/MS 8040 system.

LC-MS/MS 8040 conditions were optimized to obtain maximal sensitivity. The probe voltages (capillary voltage) for BBR and palmatine were fixed at −31.0 V and −29.0 V, respectively. Liquid nitrogen (purity >99.99%) was used as the source of nebulizer gas (1.5 L/min) and drying gas (curtain gas) (3.0 L/min). Interface voltage was −4.5 kV. Analytes were quantified in multiple reactions monitoring (MRM) mode. Selective detection ion pairs (m/z) to quantity were 336.3→320.0 for BBR, 352.3→336.1 for internal standard. The injection volume was 10 µl.

### Preparation of Tissue Samples

Each tissue sample, obtained from section 2 (Animals), was washed with saline and dried. After weighing, they were homogenized with 3 times volumes [v (µl)/w (g)] of saline. Sodium hydroxide solution (0.5 M) and ethyl ether were added to the same homogenate in a ratio of 1∶1.5 or 1∶4.5 (v/v), respectively. Samples were centrifuged at 7000 r/min for 10 min, and organic phase was taken out and evaporated to dryness in a 40°C water bath by rotary evaporator. The residues of tissue extraction were dissolved and vortex-mixed for 1 min with the mobile phase (100 µl) in a ratio of 0.5∶100 [w (g)/v (µl)]. One hundred µl of mobile phase was added when tissue weight was no more than 0.5 g. Each tissue solution was then centrifuged (12000 r/min for 5 min), and the supernatant was passed through a 0.22 µm RCF filter. An aliquot of 90 µl filtrate of each tissue sample and 10 µl internal standard was mixed by a vortex for next analyzing.

### Identification of BBR Metabolites in Tissues

The samples from section 7 (Preparation of tissue samples) were analyzed by LC/MS^n^-IT-TOF to identify BBR and its metabolites in different tissues, including liver, kidneys, muscle, lungs, brain, heart, pancreas and fat. A volume of 10 µl was injected into LC/MS^n^-IT-TOF system. For IT-TOF analysis, an ESI resource with positive mode was used, and other parameters were listed as follows: CDL temperature, 200°C; Heat block temperature, 200°C; Detector voltage, 1.70 kV; Nebulizing gas, 1.5 L/min; Collision energy, 35–50%; Drying gas pressure, 130 kPa. For full-scan MS analysis, the spectra were recorded in the range of m/z 130–1000. Mass numbers were calibrated by external standard method. The structures of BBR and its metabolites were characterized by the abundant information of the fragmentation in multiple-stage mass spectrum and accurate molecular weight provided by LC/MS^n^-IT-TOF. Berberrubine, thalifendine, demethylenberberine and jatrorrhizine standards were also used to confirm the metabolites found *in vivo.*

### Tissue Distribution of BBR and its Metabolites in Rats

Animal experiments and preparation of tissues were performed as Section 7 (Preparation of tissue samples). A Shimadzu LC-MS/MS 8040 was applied for separation and determination of BBR samples for tissue distribution. MS/MS conditions were the same as the one used in plasma (see section 5 Methodology validation). Based on the literature of possible metabolites of BBR, four Phase I metabolites were determined as the biomarkers in tissue distribution and the selective detection ion pairs (m/z) were listed as below: 336.3→320.0 for BBR, 322.11→307.08 for thalifendine (M1) and berberrubine (M2), 324.12→309.09/324.12→280.09 for demethyleneberberine (M3), and 338.13→323.11/338.13→294.11 for jatrorrhizine (M4). Selective detection ion pair (m/z) for palmatine, the internal standard, was 352.3→336.1. The peak areas of BBR and its metabolites of each tissue and internal standard were recorded. Methodology validation of BBR, including selectivity, matrix effect, and extraction recovery, in tissues was performed. The validation results of assays were similar to those of plasma and met the criteria for quantitative determinations in biological samples provided by SFDA. Moreover, the structure and physicochemical properties of metabolites were alike in those of BBR, presenting similar mass signal response. Therefore, the concentrations of BBR and its metabolites in tissues were measured based on the method of the determination in plasma. By using calibration curve obtained from section 6 (Pharmacokinetic study of plasma in rat) (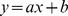), the concentrations of BBR and its metabolites (*x* (ng/g)) in tissues were calculated via the formula below:_
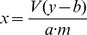
_ (*y*: peak area ratios of the BBR or metabolite to internal standard; *V* (ml): volumes of residues reconstitution solution; *m* (g): the weights of each tissue).

### Statistical Analysis

Statistical analysis was performed using GraphPad Prism® 5 software (GraphPad Software, San Diego, CA). To determine the pharmacokinetic parameters of BBR, the concentration–time data were analyzed by non-compartmental methods using DAS 2.0. *Cmax* and *Tmax* values were obtained directly from the observed concentration versus time data. Data are expressed as mean ±standard deviation (S.D.). One-way ANOVA was performed to determine statistical significance between AUC _(0-t)_ of BBR and its metabolites in each tissue and AUC _(0-t)_ of BBR in plasma. P-values equivalent to a significance level of 0.05 were considered statistically significant.

## Results and Discussion

### Methodology Optimization and Validation

In this study, LC/MS^n^-IT-TOF combined with triple quadrupole HPLC-MS/MS was first used for the qualitative and quantitative analysis of BBR and its metabolites in the investigation of tissue distribution. Through analysis of *m/z* values of fragments in multistage fragmentation for metabolites provided from LC/MS^n^-IT-TOF, metabolites of BBR were identified and characterized in chemical structures. Meanwhile, triple quadrupole LC-MS/MS was applied for quantitative study.

For the methodology validation of LC-MS/MS, all samples were found to be free of interferences with the compounds of interest. Representative MRM chromatograms of BBR were shown in Fig.1B. The retention times of BBR, M1, M2, M4 and Internal Standard were 3.6 min, 2.9 min, 3.3 min, 2.8 min and 3.6 min, respectively. The regression equation had a good linearity and was listed as below: *y* = 0.081*x*+0.0406 (*r* = 0.9991, n = 6). The lower limit of quantification (LLOQ) of the assay was at 0.05 ng/ml. The QC samples were prepared by spiking the blank rat serum (100 µl) with internal standard solution (10 µl, 250 ng/ml) of the appropriate working solutions to yield the following concentrations of BBR as high, middle and low QC samples: 0.1, 1, and 10 ng/ml. The relative standard deviation (RSD) of inter-day precision of high, middle and low QC samples were 8.10%, 11.03% and 1.79%, respectively, and the values of RSD of intra-day precision were 9.96%, 14.76% and 6.60%, respectively. The values of inter-day accuracy of high, middle and low QC samples were −11.57%, −7.97% and 1.69%, respectively and the results of intra-day accuracy were −5.16%, 2.08% and −4.15%, respectively. Data above demonstrated that the precision and accuracy of this assay were acceptable. The extraction recovery (%) displayed acceptable results with 54.80±0.49, 63.62±0.85 and 62.82±0.20 for high, middle and low QC samples, respectively. No matrix effect was observed, indicating that the extracts could not influence the detection of BBR (data are presented in Table S1 and typical MRM chromatograms were presented in Fig.S1). Stability of BBR showed to be acceptable (data are presented in Table S2). The validation results of assays met the criteria for quantitative determinations in biological samples.

Different compositions of mobile phase, including ammonium acetate, formic acid, acetic acid, were optimized and a mixture of formic acid (0.5%) and acetonitrile was determined as mobile phase. Meanwhile, the concentration of formic acid played an important role to achieve good mass behavior (both peak shape and resolution) and appropriate ionization in the MRM acquisition. The molecular ions ([M]^+^) for BBR and internal standard were the most abundant ions in the positive ion scan mode. Selective detection ion pairs (m/z) for quantification were 336.3→320.0 for BBR and 352.3→336.1 for internal standard, respectively. Palmatine was selected as internal standard for its similarity with BBR in structure and mass spectrographic behavior.

According to reports [9], there was no other method superior to that of LLOQ and range of linearity at 0.0505 ng/ml and 1.01–505 ng/ml for quantifying BBR, respectively. The method used in this study made progress in range of linearity, which was widened from 500-folds (1.01–505 ng/ml) to 1000-folds (0.05–50 ng/ml), while the value of LLOQ was still kept at high sensitivity with 0.05 ng/ml. This method could be used to quantify BBR and every metabolite within 4 min with high accuracy. As a result, the established method was more practical for simultaneously quantify the lower and higher concentration of BBR in the plasma and in the tissues, respectively.

### Identification of BBR and its Metabolites in the Tissues in Rats

After oral administration, there were three Phase I metabolites (M1, M2 and M4) found in most of tissues as well as no demethyleneberberine (M3) and phase II metabolites detected (Fig. 1C). Since the liver sample (at 8 h) would be rich in the possible metabolites, the major metabolites were identified and clarified from the sample above with data provided by LC/MS^n^-IT-TOF, subsequently structure elucidation of metabolites had been performed including thalifendine (M1), berberrubine (M2), and jatrorrhizine (M4). The same metabolites (M1, M2, and M4) were detected in the kidneys and lungs. Trace berberrubine (M2) was characterized in some organs, such as muscle, pancreas, and heart. BBR, as the parent, was found in all tissues within 48 h, whereas there were no metabolites observed in the brain and fat, indicating that BBR could permeate blood brain barrier.

M1 and M2 were a pair of isomers with the same [M]^+^ m/z being 322.1076, which was 14 Da (-CH_2_) less than that of BBR, and the same fragment of [M-CH_3_]^+^ m/z being 307.0825 (−15 Da). The loss of a CO from the MS^2^ ion of 307.0825 formed a fragment at m/z 279.0884 (MS^3^), which further produced fragment at 263.0567 (−16 Da) on MS^4^. Ion m/z 263.0567 could lose 58 Da to form fragment m/z 205.0493 on MS^5^. Chemical structures of M1 and M2 were further confirmed with standards. [M]^+^ m/z of M4 was 338.1384 and the MS^2^ spectrum had fragment of m/z 323.1146 (−15 Da), indicating a loss of CH_3_ from molecular ion. It can further fragment to 307.0793 (-CH_3_-CH_3_-H), 294.1093 (-CH_3_-H-CO) and 279.0868 (-CH_3_-H-CO-CH_3_) on MS^3^ and MS^4^ stages, respectively. The structure was also confirmed by comparing with standard, and it should be jatrorrhizine (formula, mass errors of MS^1^ and MS^n^ data from BBR and its metabolites are presented in Table S3).

Therefore, metabolites M1, M2 and M4 were selected and determined as the biomarkers in the biological samples for the next distribution study.

### Pharmacokinetics and Tissue Distribution of BBR

The pharmacokinetics of oral BBR was studied, and tissue distribution of BBR at ten different time points (0, 0.25, 0.5, 1.0, 2, 4, 8, 12, 24 and 48 h) were quantified in the liver, kidneys, muscle, lungs, brain, heart, pancreas and fat.

**Figure 2:**
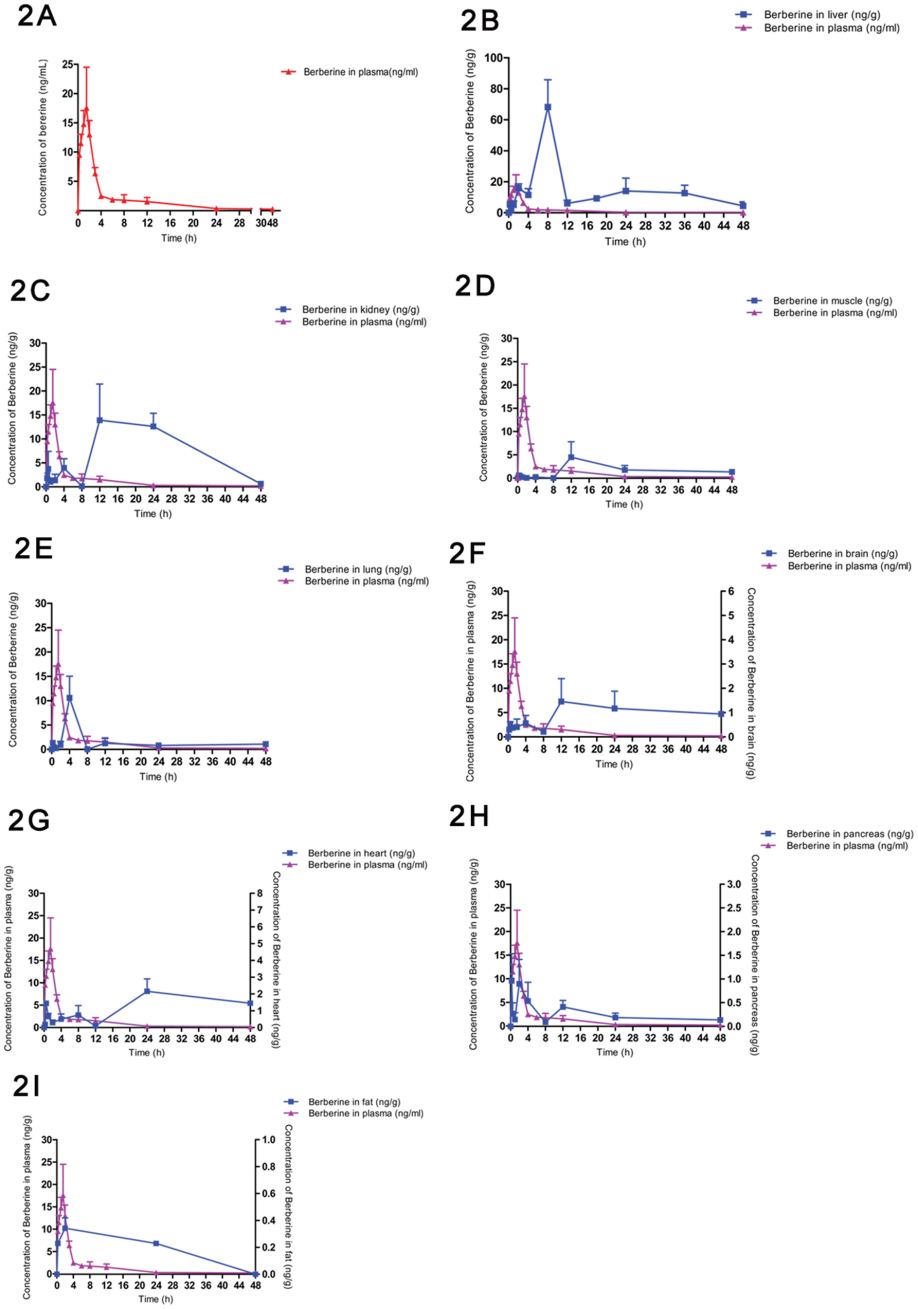
Tissue distribution of BBR. 2A Plasma concentration-time profile of BBR after oral administration of 200 mg/kg in 48 h (n = 6) 2B Profiles of BBR in liver distribution in SD rats after oral administration of 200 mg/kg (n = 6) 2C Profiles of BBR in kidney distribution in SD rats after oral administration of 200 mg/kg (n = 6) 2D Profiles of BBR in muscle distribution in SD rats after oral administration of 200 mg/kg (n = 6) 2E Profiles of BBR in lung distribution in SD rats after oral administration of 200 mg/kg (n = 6) 2F Profiles of BBR in brain distribution in SD rats after oral administration of 200 mg/kg (n = 6) 2G Profiles of BBR in heart distribution in SD rats after oral administration of 200 mg/kg (n = 6) 2H Profiles of BBR in pancreas distribution in SD rats after oral administration of 200 mg/kg (n = 6) 2I Profiles of BBR in fat distribution in SD rats after oral administration of 200 mg/kg (n = 6).

The calculation of BBR and its metabolites distributed in tissues was based on the results of BBR concentration in plasma. The pharmacokinetic profile was represented in Fig.2A and Table 1. According to the data of pharmacokinetics software, the values of *t*_1/2_ and *T*_max_ were 14.73±7.28 h and 1.33±0.29 h, respectively. The results of AUC _(0-∞)_ and AUC _(0-t)_ were 86.37±13.57 ng/mL×h, and 75.83±5.60 ng/mL×h, respectively. The value of *C*_max_ was 25.85±7.34 µg/L. In the meantime, abundance of BBR in different tissues was compared with that in plasma within 48h. Profiles were sorted by amounts of BBR in tissues from most to least (Fig.2B–2I).

**Table 1:** Pharmacokinetic parameters of BBR after oral administration (200 mg/kg).

Sample	*t*_1/2_ (h)	AUC _(0-∞)_ (ng/mL×h)	AUC _(0-t)_ (ng/mL×h)	*C*_max_ (µg/L)	*T*_max_ (h)
BBR	14.73±7.28	86.37±13.57	75.83±5.60	25.85±7.34	1.33±0.29

Data are represented as mean ±S.D. (n = 6).

Results indicated that BBR was widely distributed in the tissues, and the concentrations of BBR in certain tissues were much higher than that in the plasma. BBR was detected in liver, kidneys, muscle, lungs, brain, heart, pancreas and fat in a descending order of distributed amounts within 48h. The pharmacokinetic profile suggested that BBR reached the tissues within 0.25 h (the earliest time point for sampling) after dosing and its level in most of the investigated tissues was higher (or much higher) than that in the plasma at 4 h after administration. As shown in Table 2 and Fig.2B–2I, BBR had a significant distribution in the liver with an AUC _(0-t)_ value of 728.6±188.1 ng/mL×h and reached maximum concentration of BBR in liver 8 h after treatment. The abundance of BBR in other tissues was listed as below based on the AUC _(0-t)_ calculation: kidneys >muscle >lungs >brain >heart >pancreas >fat. Time to the maximum concentration in tissues varied with 2 h for pancreas and fat, 4 h for lungs, 12 h for kidneys, muscle, and brain, 24 h for heart.

**Table 2:** AUC _(0-t)_ values of tissues and tissue/plasma ratios of BBR and its metabolites.

Tissues	liver	kidney	muscle	lung	brain	heart	pancreas	fat
BBR AUC _(0-t)_ of tissues (ng/mL×h)	728.6±188.1^a^	362.7±63.5^a^	84.2±15.3	66.0±10.2	47.8±6.4	47.4±5.6	12.0±2.0	6.8±1.1
Metabolites AUC_(0-t)_ of tissues (ng/mL×h)	2103.5±347.2^b^	161.4±29.8	6.2±1.9	31.4±7.3	ND*	0.6±0.18	0.3±0.03	ND
BBR AUC _(0-t)_ of tissues/BBR AUC _(0-t)_ of plasma	10.17±2.7	5.06±0.9	1.18±0.3	0.92±0.2	0.67±0.1	0.66±0.1	0.17±0.03	0.09±0.02
Metabolites AUC_(0-t)_ of tissues/BBR AUC _(0-t)_ of plasma	29.36±4.9	2.25±0.5	0.09±0.03	0.44±0.1	ND	0.008±0.01	0.004±0.0003	ND

* ND: not detected. Data are represented as mean ±S.D. (n = 6). One-way ANOVA: BBR AUC _(0-t)_ or Metabolites AUC _(0-t)_ of tissues were compared with control (AUC_(0-t)_ of plasma), which was 71.65 (ng/mL×h).

a p<0.0001(which means “***” in the result shown by GraphPad Prism® 5 software), compared BBR AUC _(0-t)_ of tissues with BBR AUC_(0-t)_ of plasma;

b p<0.0001, compared metabolites AUC _(0-t)_ of tissues with BBR AUC _(0-t)_ of plasma.

Liver was the main metabolic tissue, in which the amount of BBR was more than ten times of that in plasma. BBR peak was observed at 0.25 h, 2 h, 8 h and 24 h respectively, and its highest concentration (68.19 ng/g) was presented at 8h. BBR exhibited three peaks in kidneys as well at 0.5 h, 4 h and 12 h, respectively, with the highest concentration (13.92 ng/g) presented at 12 h. Besides liver and kidney, BBR was also detected in other tissues like muscle, lungs, brain, heart, pancreas and fat, with multiple peaks (Fig. 2D–2H). BBR remained relatively stable in the tissues for 48 h, especially with a high level in the liver. The pharmacokinetics of BBR may be associated with its wide distribution and the enterohepatic circulation of the metabolites [35]–[37].

Results of tissue distribution of BBR provided a partial explanation for the discrepancy between low blood level of BBR and its pharmacological effects. In fact, some natural products with low bioavailability (<1.0%) in oral route were reported to be active in treating patients, such as ginsenoside Rg1, Rbs and Rc from ginseng [38]–[40]. Being the main organ in which TG and cholesterol are manufactured and metabolized, liver plays a significant role in controlling blood lipid. A large amount of BBR distributed in the liver, provided evidence for its cholesterol-lowering activity [5]. Moreover, distribution of BBR in the brain and heart might be associated with BBR’s effects on CNS and cardiovascular system. BBR’s feature in organ distribution was similar to that of Tanshinone IIA, the main ingredient of a Chinese medicine Danshen, which is used for the treatment of cardiovascular disease [41].

### Tissue Distribution of BBR Metabolites

**Figure 3:**
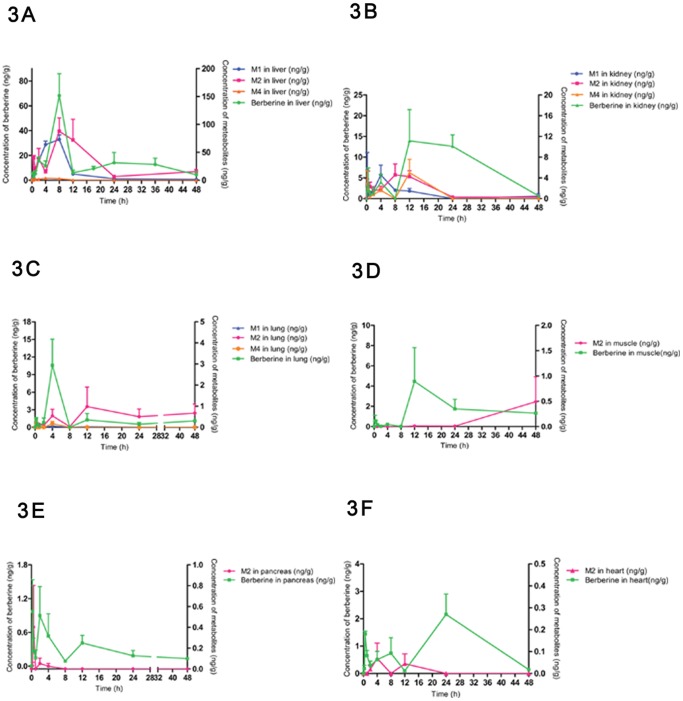
Tissue distribution of metabolites of BBR. 3A Profiles of BBR and its metabolites in liver distribution in SD rats after oral administration of 200/kg (n = 6) 3B Profiles of BBR and its metabolites in kidney distribution in SD rats after oral administration of 200 mg/kg (n = 6) 3C Profiles of BBR and its metabolites in lung distribution in SD rats after oral administration of 200 (n = 6) 3D Profiles of BBR and its metabolites in muscle distribution in SD rats after oral administration of 200 mg/kg (n = 6) 3E Profiles of BBR and its metabolites in pancreas distribution in SD rats after oral administration of 200 mg/kg (n = 6) 3F Profiles of BBR and its metabolites in heart distribution in SD rats after oral administration of 200 mg/kg (n = 6).

After oral administration, metabolites of BBR were found in the liver, kidneys, lungs, muscle, heart and pancreas. Most of the metabolites reached the tissue within 4 h after dosing. Liver was the highest carrier, followed by kidneys, lungs, muscle, heart and pancreas. All of the metabolites were Phase I metabolites, and M1 and M2 were predominated. Accordingly, abundance of metabolites in tissues was compared with that of BBR in the tissue (Fig.3A to 3F, and Table 3), in order to learn the tissue distribution of BBR.

**Table 3:** Distribution of BBR metabolites in rat.

No.	Metabolite	AUC _(0-t)_ (ng/mL×h) of important tissues					
		Liver	Kidney	Lung	Heart	Muscle	Pancreas
M1	Thalifendine	700.9±91.3	42.1±9.3	0.42±0.17	ND*	ND	ND
M2	Berberrubine	1370±292.4	71.5±10.9	30.46±8.04	0.60±0.18	6.17±1.9	0.31±0.03
M4	Jatrorrhizine	32.6±8.8	47.8±22.7	0.55±0.36	ND	ND	ND

* ND: not detected. Data are represented as mean ±S.D. (n = 6).

The volume of BBR and its metabolites was in a descending order of BBR>M2>M1>>M4. M2 (berberrubine) and M1 (thalifendine) were the major metabolites with a proportion of 65.1% and 33.3% among the metabolites detected in the liver. The value of AUC _(0-t)_ of metabolites was 2103.5±347.2 ng/mL×h in the liver within 48 h, 29 times higher than that of BBR in plasma. M1 reached its peak at 8 h after administration, and decreased quickly within 8–12 h. M2 reached its maximum concentration at 8 h as well, and decreased gradually before 24 h. The total AUC _(0-t)_ for BBR and its metabolites in the liver was nearly forty times higher than that in plasma. BBR was least detected at 12 h, suggesting that the rate of metabolism (M1, M2 and M4) was faster than that of the distribution in the liver between 8–12 h, while the volume of BBR became more and more between 12–24 h, indicating there may be an enterohepatic circulation. Between 24–48 h, BBR was decreased with increasing M2. Therefore, liver was the main metabolic tissue for BBR.

BBR concentration in the kidneys was much higher than that of its metabolites, including M1, M2 and M4. The sum of AUC _(0-t)_ values for BBR and its metabolites in the kidneys was seven times more than that of BBR in plasma. Compared with metabolites in the liver, metabolites in the kidneys was significantly less but with multiple peaks. The AUC _(0-t)_ value of metabolites within 48 h was 161.4±29.8 ng/mL×h, and was 2.25 times of parent drug in the plasma. In the kidneys, metabolites peaked to their maximum in 15–30 min, demonstrating that distribution in kidney was quite rapid. There were two other peaks at 4 h and 12 h, so there may be a redistribution similar to liver. Kidneys were the main excretion tissue for BBR.

Similarly, BBR in lungs was much more than its metabolites, including M1, M2 and M4. M2, which was in majority, was reached in the maximum concentration at 12 h. AUC _(0-t)_ level of metabolites within 48 h was 31.4±7.3 ng/mL×h, and much lower than that of BBR in plasma. Metabolites could be detected at 0.25 h in the lung, demonstrating that distribution in the lungs was also quite rapid. And then M1, M2 and M4 increased within 2–4 h and peaked at 4 h. The behavior of BBR and its metabolites in the lung was very similar to the one in kidneys, meaning rapid distribution and redistribution.

In the muscle, BBR was also the one with higher level; only M2 was detected and tended to increase with time. The AUC _(0-t)_ of metabolites within 48 h was 6.2±2.1 ng/mL×h, and significantly lower than that of BBR. M2 level in the pancreas and heart was very low, and no metabolite was detected in the brain or fat.

Recent research has reported that thalifendine (M1) and berberrubine (M2) are the two major active metabolites, which up-regulate InsR expression as well as LDLR and activate AMPK, although the potency was largely reduced. For LDLR mRNA expression, M1 and M2 remained to be active with about 30% of the BBR activity. Therefore, Phase I metabolites of BBR could be an important part to understand the BBR pharmacodynamics and pharmacokinetics.

## Conclusion

BBR and its bioactive metabolites were detected in the liver, kidneys, muscle, lung, brain, heart, pancreas and fat after oral administration. The concentration of BBR and the metabolites in the organs was often higher or much higher than that in blood. A dominant tissue distribution of BBR and its metabolites were observed in the liver, which is the most important organ for energy metabolism. This explains at least partially the BBR’s effect on cholesterol, glucose, triglyceride in patients. BBR is also distributed in the brain and heart, which explains its effects on CNS and cardiovascular system. Besides, BBR and its metabolites remain relatively stable in the tissues. These pharmacological results help us to understand why BBR is active *in vivo,* even if its blood concentration is low.

## Supporting Information

Figure S1 **Typical MRM chromatograms of BBR in plasma of rats.** A: MRM chromatographs of blank matrix (plasma of rats) with I.S., for detection of BBR (a) and I.S. (b); B: MRM chromatographs of blank matrix with BBR and I.S., for detection of BBR (a) and I.S. (b); C: MRM chromatographs of plasma sample which was collected from rats with oral BBR (200mg/kg), for detection of BBR (a) and I.S. (b).
(TIF)

Table S1 **Matrix effect of BBR in plasma of rats.**
(DOC)

Table S2 **Stability of BBR in plasma of rats.**
(DOC)

Table S3 **Formula, mass errors of MS^1^ and MS^n^ data from BBR and its metabolites detected by LC/MS^n^-IT-TOF in the tissues in rats.**
(DOC)
