# Supplementary figures and images for: Tissue Distribution of Berberine and Its Metabolites after Oral Administration in Rats
Source: PLoS One. 2013 Oct 31;8(10):e77969. doi: 10.1371/journal.pone.0077969 (PMC3815028; doi:10.1371/journal.pone.0077969)

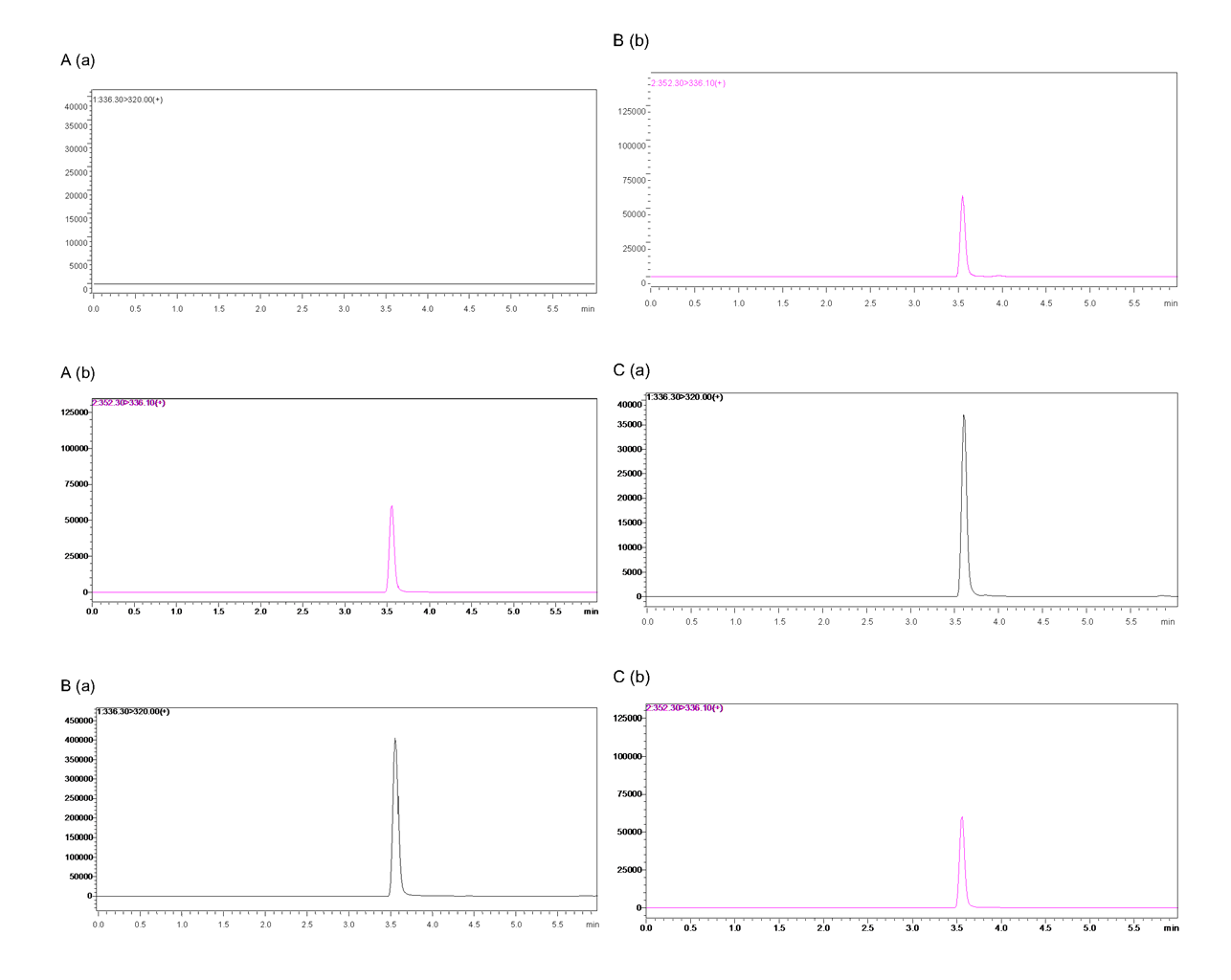

Supplement: Figure S1 — Typical MRM chromatograms of BBR in plasma of rats. A: MRM chromatographs of blank matrix (plasma of rats) with I.S., for detection of BBR (a) and I.S. (b); B: MRM chromatographs of blank matrix with BBR and I.S., for detection of BBR (a) and I.S. (b); C: MRM chromatographs of plasma sample which was collected from rats with oral BBR (200mg/kg), for detection of BBR (a) and I.S. (b). (TIF) [file pone.0077969.s001.tif]
