# Supplementary material for: Tissue Distribution of Berberine and Its Metabolites after Oral Administration in Rats
Source: PLoS One. 2013 Oct 31;8(10):e77969. doi: 10.1371/journal.pone.0077969 (PMC3815028; doi:10.1371/journal.pone.0077969)
Supplement: Table S1 — Matrix effect of BBR in plasma of rats. (DOC) [file pone.0077969.s002.doc]

**Table S1 Matrix effect of BBR in plasma of rats**

| Concentration of BBR in theory（ng/mL） | 0.1 | 1 | 10 |
| --- | --- | --- | --- |
| Matrix effect (%) | 76.53±32.17 | 92.14±10.10 | 93.82±8.23 |

Data are represented as mean ±S.D. (*n* = 5)
