# Supplementary material for: Tissue Distribution of Berberine and Its Metabolites after Oral Administration in Rats
Source: PLoS One. 2013 Oct 31;8(10):e77969. doi: 10.1371/journal.pone.0077969 (PMC3815028; doi:10.1371/journal.pone.0077969)
Supplement: Table S2 — Stability of BBR in plasma of rats. (DOC) [file pone.0077969.s003.doc]

**Table S2 Stability of BBR in plasma of rats**

| Concentration of BBR in theory（ng/mL） | 0.1 | 1 | 10 |
| --- | --- | --- | --- |
| Room temperature for 24hrs | -19.07 | -1.83 | 3.97 |
| 4 ^o^C for 24hrs | -13.11 | -6.31 | 13.82 |
| Freezing-thaw for 3 times | -17.90 | 13.18 | -10.33 |

Data are represented as mean ±S.D. (*n* = 5)
