# Supplementary material for: Tissue Distribution of Berberine and Its Metabolites after Oral Administration in Rats
Source: PLoS One. 2013 Oct 31;8(10):e77969. doi: 10.1371/journal.pone.0077969 (PMC3815028; doi:10.1371/journal.pone.0077969)
Supplement: Table S3 — Formula, mass errors of MS1 and MSn data from BBR and its metabolites detected by LC/MSn-IT-TOF in the tissues in rats. (DOC) [file pone.0077969.s004.doc]

**Table S3 Formula, mass errors of MS^1^ and MS^n^ data from BBR and its metabolites detected by LC/MS^n^-IT-TOF in the tissues in rats**

| Compound | t_R_ (min) | Formula (M) | Fragments | | | | |
| --- | --- | --- | --- | --- | --- | --- | --- |
|  |  |  | MS^1^[M]^+^ | MS^2^ m/z | MS^3^ m/z | MS^4^ m/z | MS^5^ m/z |
| BBR | 10.71 | C_20_H_18_NO_4_ | 336.1193 | 292.0939, 321.0952, 275.0937 | 277.0688, 249.0760, 234.0882 | 248.0689, 219.0630 | — |
| M1 | 9.78 | C_19_H_16_NO_4_ | 322.1076 | 307.0825, 279.0874 | 279.0884 | 263.0567, 220.0749 | 205.0493, 234.0394, 178.0643 |
| M2 | 10.10 | C_19_H_16_NO_4_ | 322.1053 | 307.0829, 279.0852 | 279.0852, 250.0824 | 263.0673, 222.0919 | 205.0486, 234.0518, 178.0586 |
| M4 | 9.63 | C_20_H_20_NO_4_ | 338.1384 | 294.1129, 323.1146, 322.1073 | 307.0793, 294.1093, 279.0873 | 279.0868, 251.0922 | 250.0825, 222.0817 |
